# Sediment accretion and nutrient enrichment enhance the growth and vegetative propagation of *Phalaris arundinacea* growing within a *Carex thunbergii* stand

**DOI:** 10.3389/fpls.2024.1459663

**Published:** 2024-10-14

**Authors:** Xin-zhi Guo, Jia-shun Zhong, Wen-jing Sun, Xiang-rong Song, Jing Liu, Xin-sheng Chen

**Affiliations:** ^1^ School of Resources and Environmental Engineering, Anhui University, Hefei, China; ^2^ Anhui Shengjin Lake Wetland Ecology National Long-term Scientific Research Base, Dongzhi, China; ^3^ Anhui Province Key Laboratory of Wetland Ecosystem Protection and Restoration, Anhui University, Hefei, China; ^4^ Baiyangdian Basin Ecological Environment Monitoring Center, Baoding, China

**Keywords:** *Carex* grasslands, floodplain wetlands, macrophyte distribution, sediment deposition, vegetative propagation

## Abstract

Sediment accretion (burial) and nutrient enrichment may exert a synergistic influence on the growth and distribution of macrophytes in floodplain wetlands; however, this phenomenon has rarely been examined. In this study, we investigated the effects of sediment accretion and nutrient enrichment on the growth and vegetative propagation of *Phalaris arundinacea* within a *Carex thunbergii* stand (one *P. arundinacea* ramet within 25 C*. thunbergii* ramets) using a factorial sediment burial (0, 3, and 6 cm) and nutrient addition (low, medium, and high) experimental design. High sediment burial (6 cm) without nutrient addition decreased the aboveground and total biomass of *C. thunbergii* but did not affect *P. arundinacea*, indicating that *P. arundinacea* is more tolerant to sediment burial than *C. thunbergii*. Moderate sediment burial (3 cm) with nutrient addition enhanced the aboveground and total biomass of *P. arundinacea* but did not affect *C. thunbergii*, indicating that *P. arundinacea* may gain a growth advantage over *C. thunbergii* under moderate sedimentation. High sediment burial with nutrient addition increased the number of rhizomes and ramets produced by *P. arundinacea* but did not affect *C. thunbergii*, indicating that the relative abundance of *P. arundinacea* may increase within the *Carex* community under high sedimentation conditions. Based on these results, it can be concluded that an increased sedimentation rate facilitates the invasion of *P. arundinacea* into *Carex* grasslands, and this invasion is further enhanced by nutrient enrichment. Therefore, management measures should be taken to reduce the sediment load and nutrient input to prevent *Carex* grasslands invasion by *P. arundinacea* and maintain the ecological function of floodplain wetlands.

## 1 Introduction

Macrophyte vegetation in floodplain wetlands plays crucial ecological roles such as controlling sediment nutrients and providing food sources and habitats for wildlife (Dar et al., 2020; Dou et al., 2023). Macrophytic species are typically distributed along hydrological gradients and exhibit dynamic zonal patterns (Zhang et al., 2020). Hydrological conditions and sediment properties influence their growth and distribution, thereby influencing the ecological function of floodplain wetlands (Chen et al., 2014; Liu et al., 2018; Zhang et al., 2020; Jing et al., 2021; Moritz et al., 2022).

Flood regimes and sediment properties have changed considerably in many freshwater environments because of climatic changes and anthropogenic activities, which may influence the distribution patterns of macrophytes (Blakey et al., 2017; Chen et al., 2018; Ji et al., 2021; Huang et al., 2022). In floodplain wetlands, sedimentation co-occurs with flooding (Maun, 1998). After flooding, sediments are deposited on floodplain wetlands, ranging in depth from a few millimeters to several centimeters per year (Ou et al., 2019; Baniya et al., 2020; Geng et al., 2021; Kretz et al., 2021; Huang et al., 2022; Zeng et al., 2023). Wetland macrophytes with different growth forms respond differently to sediment accretion and have evolved various morphological and physiological strategies to acclimate to sediment burial stress (Li et al., 2015). Caulescent macrophytes, such as *Phalaris arundinacea* and *Polygonum hydropiper*, elongate the internode length, release dominant buds on their stems, and increase stem biomass to escape sediment accretion (Chen et al., 2014, 2017a). Non-caulescent macrophytes, such as *Carex brevicuspis* and *Vallisneria natans*, may not respond as successfully to sediment accretion as caulescent macrophytes (Pan et al., 2012). Thus, the differential responses to sediment accretion among macrophyte species may alter macrophyte distribution (Owen et al., 2004).

Nutrients in floodwater, such as nitrogen and phosphorus, may be retained in the sediment after flooding, facilitating the acclimation of wetland macrophytes to sediment stress (Ou et al., 2019; Huang et al., 2022; Zeng et al., 2023). Nutrients in the sediment may play an important role in stimulating plant growth following sediment burial. However, their effects on macrophyte growth vary from positive to negative, particularly following deep sediment burial (Chen et al., 2017b). Therefore, the effects of sediment nutrient enrichment on growth and vegetative reproduction may differ among macrophyte species.

Moreover, sediment accretion and nutrient enrichment are concurrent processes that may exert a synergistic influence on the growth and distribution of macrophytes but have rarely been examined. In this study, we investigated the effects of sediment accretion and nutrient enrichment on the growth and propagation of *Phalaris arundinacea* in a *C. thunbergii* stand. *Carex* is the dominant species in the floodplain wetlands of the Yangtze River basin, with *P. arundinacea* sparsely distributed within the *Carex* vegetation (Jing et al., 2017). Recently, the abundance and distribution of *P. arundinacea* has increased, especially in lacustrine wetlands with considerable sedimentation (Nelson and Anderson, 2015). Sediment accretion and nutrient enrichment are potential drivers of these changes, although empirical evidence supporting this is limited. We tested two hypotheses: (1) *P. arundinacea* can tolerate higher sediment accretion than *C. thunbergii* because it can escape sediment burial more efficiently than *C. thunbergii*, and (2) *P. arundinacea* may benefit more from nutrient enrichment than *C. thunbergii* because it can utilize nutrients more efficiently than *C. thunbergii* (Wetzel and van der Valk, 1998; Nelson and Anderson, 2015).

## 2 Materials and methods

### 2.1 Study site

Shengjin Lake (30°15′-30°30′N, 116°55′-117°15′E), located in the lower reaches of the Yangtze River, was listed as an internationally important wetland in 2015 (Wang et al., 2018). Influenced by the subtropical monsoon climate, wetlands tend to be inundated from June to October and are exposed from November to May. *Carex thunbergii* is the dominant plant community in the water-level fluctuation zone of this wetland.

### 2.2 Study species

*Carex thunbergii* is a rhizomatous sedge widely distributed in lakes and wet grasslands of eastern Asia (Dai et al., 2010). The pseudoculms of *C. thunbergii* have overlapping sheaths and are usually 40–100 cm high. *Carex thunbergii* often forms a monodominant community with a coverage of approximately 100% (Jia et al., 2020). The aboveground shoots of *C. thunbergii* are submerged and decompose during the flooding season, and new shoots emerge from the belowground rhizome buds immediately after the floodwaters recede (Chen et al., 2014).

*Phalaris arundinacea* is widely distributed in riparian and lacustrine wetlands in the subtropical and temperate regions of the Northern Hemisphere (Wu and Phillips, 2006). The erect culms are reed-like and 60–150 cm in height. They produce extensively spread rhizomes, enabling them to reproduce vigorously and spread aggressively (Wu and Phillips, 2006; Chen et al., 2017a). In the *Carex* community, *P. arundinacea* is a commonly observed companion species; however, its relative abundance and importance have increased considerably in recent times, which may affect the structure and function of *Carex* grasslands (Chen et al., 2017a).

### 2.3 Experimental design

**Figure 1:**
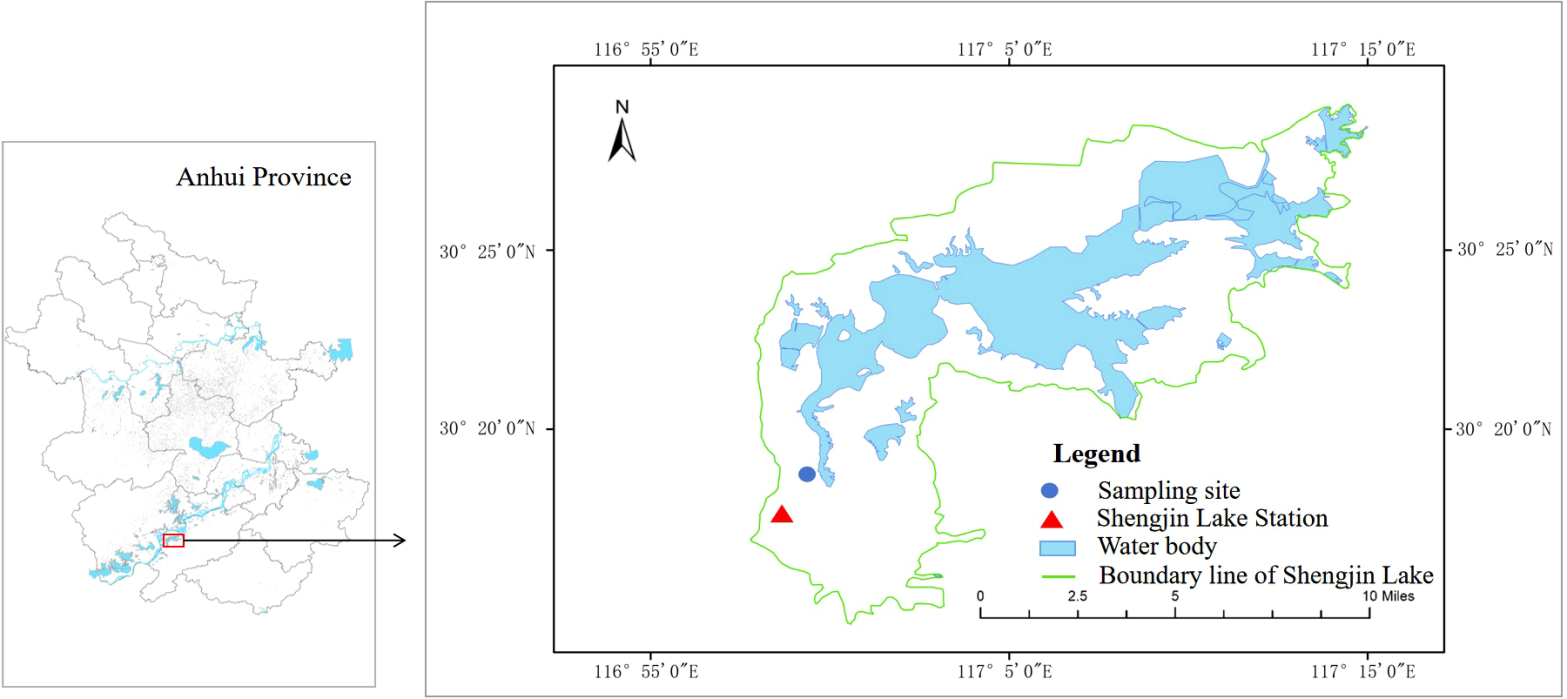
Study area and locations of sampling sites in Shengjin Lake, China.

The experiment was conducted at the Shengjin Lake Station for Wetland Ecosystem Research, Dongzhi County, Anhui Province, China (**Figure 1**). Before the experiment, we surveyed the ramet density of *P. arundinacea* in *Carex* grasslands in Shengjin Lake. The ramet density of *P. arundinacea* ranges from 1 to 112 ramets m^-2^, and density of *C. thunbergii* ranges from 400 to 2240 ramets m^-2^. Then, young ramets with soil from the Yaozui section of the lake were dug up and transported to the research station on April 3, 2023. Ramets were cultivated in a nursery bed containing a 10 cm soil/sand mixture (1:1 v/v) sourced from the Shengjin Lake. On April 10, one *P. arundinacea* ramet and 25 C*. thunbergii* ramets of similar heights (4–6 leaves and 14–20 cm in height) were planted in plastic buckets containing wetland soil 15 cm deep (each plastic bucket was 20 cm in diameter and 30 cm in height). There are many small holes (< 3 mm) at the bottom of the buckets, which allowed water to penetrate. The densities of *C. thunbergii* and *P. arundinacea* in the experiment were 796 and 32 ramets m^-2^, respectively, which were within the density range observed in the field.

**Figure 2:**
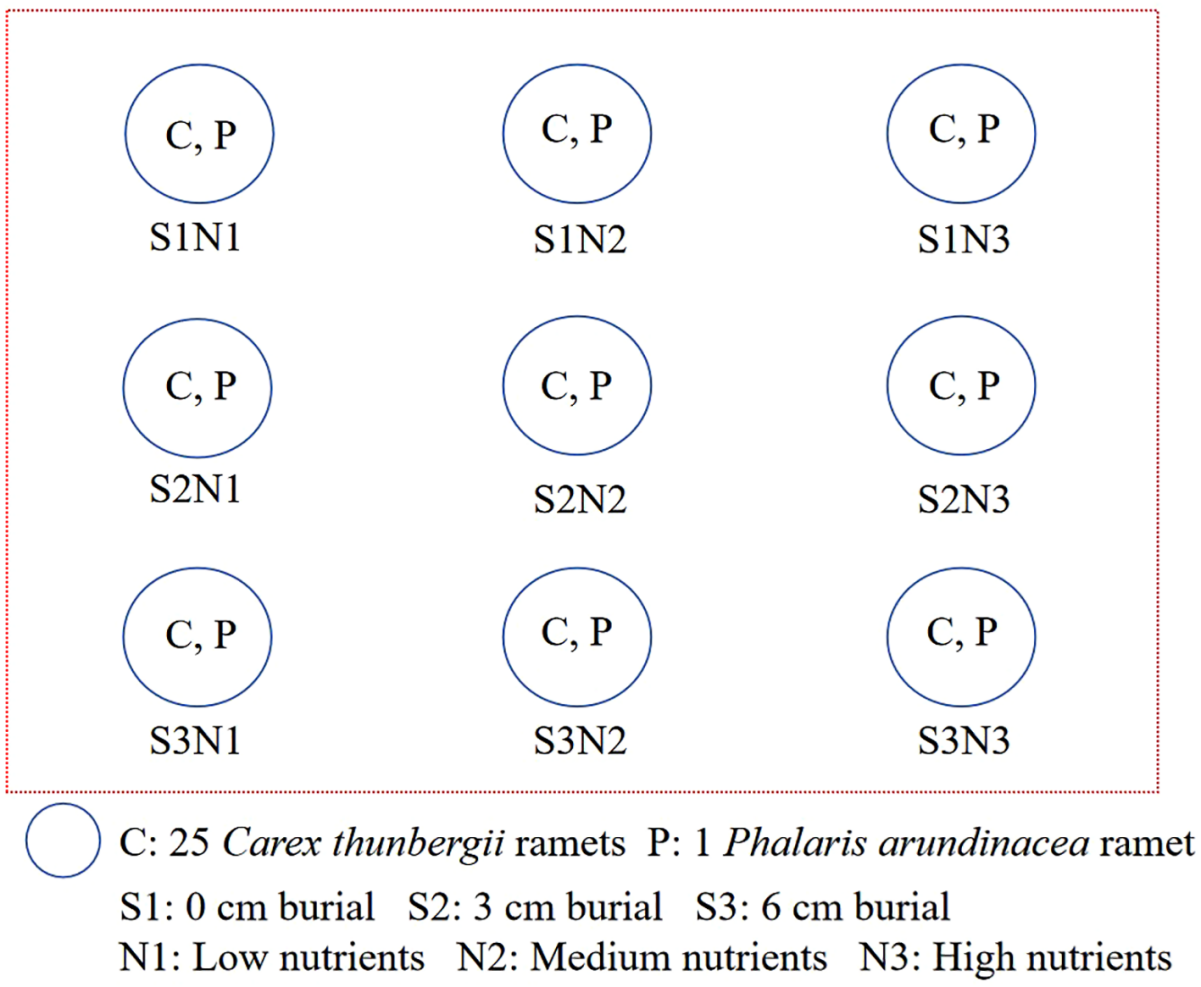
Schematic plan of the experimental design.

The experimental design was a randomized block with five replicates (**Figure 2**). The experiment was conducted in separate outdoor water tanks (98 × 76 × 68 cm). Three sediment burial depths (0, 3, and 6 cm) and three levels of nutrient addition (low, medium, and high) were used in the experiment. The low-nutrient sediment was sand collected from Shengjin Lake, which contained 0.03 mg kg^-1^ total nitrogen, 0.02 mg kg^-1^ total phosphorus, and 0.48 mg kg^-1^ total potassium. The medium- and high-nutrient sediments were created by mixing lake sand homogeneously with 1 or 2 g of Osmocote slow-release fertilizer (501 Osmocote Plus [N-P-K, 15-10-12 + 2 MgO + TE ICL], Belgium N.V.). For the 3 and 6 cm sediment accretion with nutrient addition treatments, medium- or high-nutrient sand was added to the container to the corresponding depth. For the 0 cm sediment accretion with nutrient addition treatment, 1 or 2 g of slow-release fertilizer was added to the soil surface and covered with a thin layer of sand (< 0.2 cm). The water level in the tanks was maintained at 15 cm (0 cm for each plant) during the experiment. Plants were monitored weekly, and new ramets were marked with plastic labels.

### 2.4 Harvest and measurement

Plants were harvested on August 29, 2023, 20 weeks after treatment. The plants were carefully removed from the plastic containers to ensure the integrity of the aboveground and belowground parts. The plants were washed with tap water to remove attached sediment. Ramet height, root length, and number of ramets and rhizomes of each *P. arundinacea* parent ramet were measured. We also measured the number of rhizomes and ramets produced by each *C. thunbergii* parent ramet in the plastic containers. We then selected five *C. thunbergii* parent ramets from each container to measure the ramet height and root length. The ramets of *P. arundinacea* and *C. thunbergii* were separated into roots, shoots, and leaves. The biomass of each plant part was measured after drying at 85°C for 48 h in an oven.

### 2.5 Data analysis

Two-way ANOVA was performed to evaluate the main effects and interactions of sediment burial depth and nutrient addition levels on biomass accumulation, biomass allocation, ramet height, root length, and the number of new ramets and rhizomes. Multiple comparisons of the means were performed using Tukey’s test at a significance level of 0.05. Data were log^10^-transformed, if necessary, to reduce the heterogeneity of variance, and homogeneity was confirmed using Levene’s test. All analyses were performed using the “car” and “multcomp” package in R program (v. 4.1.2; R Core Team 2020).

## 3 Results

### 3.1 Biomass accumulation and allocation

**Figure 3:**
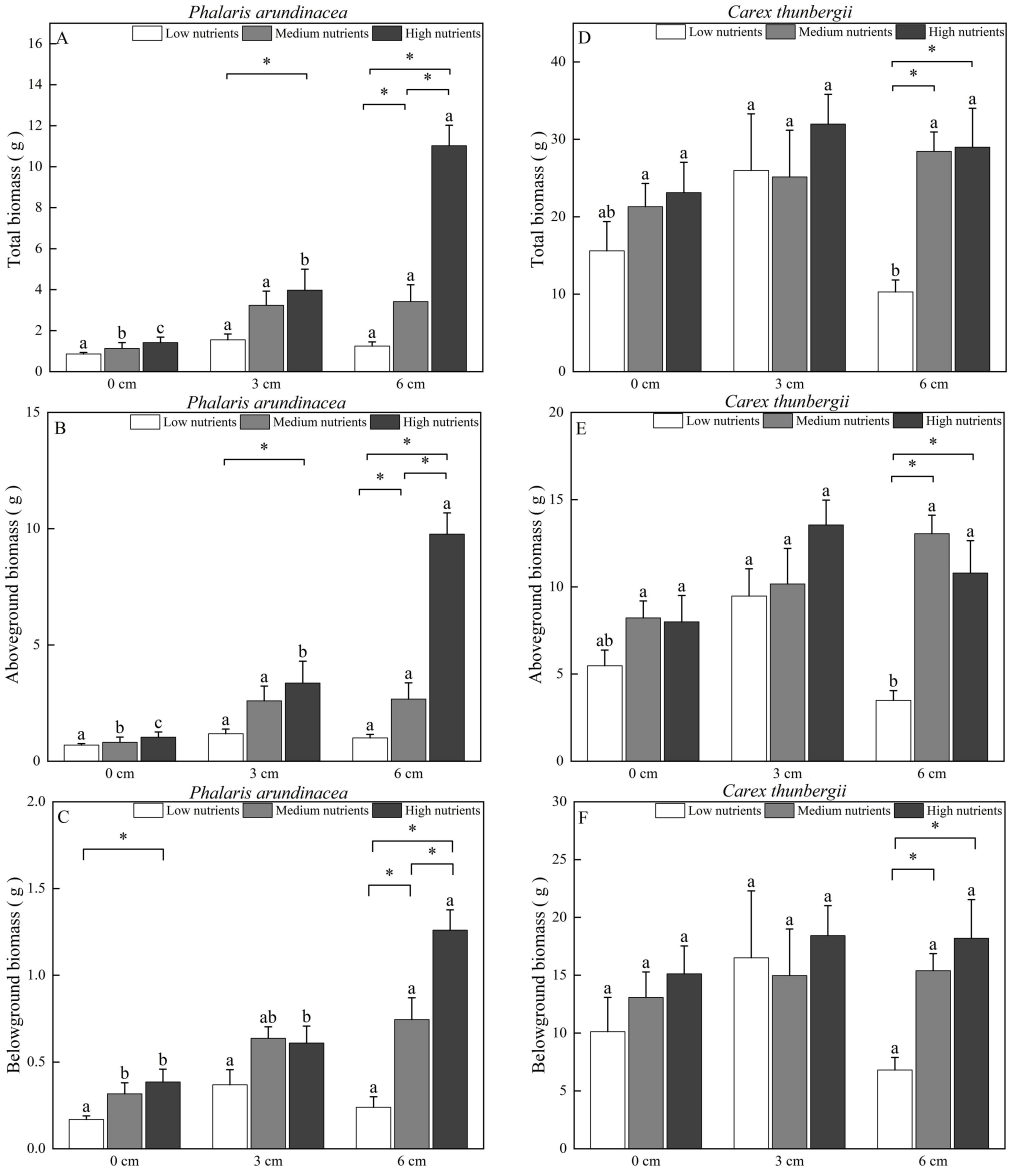
Biomass accumulation in *Phalaris arundinacea* and *Carex thunbergii* at three sediment burial depths with three nutrient levels. Total biomass, Aboveground biomass and Belowground biomass of *Phalaris arundinacea* **(A–C)**, Total biomass, Aboveground biomass and Belowground biomass of *Carex thunbergii* **(D–F)**. Different lowercase letters indicate significant differences among burial depth treatments at the same nutrient level (P < 0.05). * indicates significant differences among different nutrient level treatments at the same sediment burial depth (P < 0.05).

Aboveground, belowground, and total biomass of *P. arundinacea* were significantly affected by burial depth and nutrient levels (*P* < 0.01; **Table 1**). Sediment accretion increased the aboveground, belowground, and total biomass of *P. arundinacea* growing in medium- and high-nutrient sediments (**Figures 3A–C**). Nutrient addition increased the aboveground and total biomass of *P. arundinacea* growing in the 3 and 6 cm sediment burial treatments (**Figures 3A, B**).

**Table 1:** Summary of two-way ANOVA (*F*-values) for total biomass, aboveground biomass, belowground biomass, ramet height, root length, and number of rhizomes and ramets in *Phalaris arundinacea* and *Carex thunbergii* grown at three sedimentation depths and three nutrient levels.

	*Phalaris arundinacea*			*Carex thunbergii*		
	Burial depth (B)	Nutrient level (N)	B × N	Burial depth (B)	Nutrient level (N)	B × N
Total biomass	28.117^***^	26.301^***^	5.864^**^	2.337^ns^	4.662^*^	1.286^ns^
Aboveground biomass	28.059^***^	22.675^***^	5.937^***^	5.659^**^	10.340^***^	3.042^*^
Belowground biomass	13.392^***^	23.024^***^	2.726^*^	1.091^ns^	6.220^**^	1.369^ns^
Ramet height	14.291^***^	9.566^***^	1.807^ns^	4.715^*^	0.459^ns^	3.889^*^
Root length	5.606^**^	4.095^*^	0.998^ns^	0.592^ns^	13.204^***^	0.514^ns^
No. of rhizomes	5.283^**^	2.768^ns^	3.013^*^	3.766^*^	0.265^ns^	0.089^ns^
No. of ramets	10.088^***^	11.192^***^	5.672^**^	1.027^ns^	0.995^ns^	0.501^ns^

***P < 0.001; **P < 0.01; *P < 0.05; ^ns^ *P* > 0.05.

The total and belowground biomass of *C. thunbergii* were only affected by the nutrient levels (**Table 1**). Nutrient addition increased the total and belowground biomass of *C. thunbergii* growing in the 6 cm sediment burial treatment (**Figures 3D, F**). The aboveground biomass of *C. thunbergii* was significantly affected by burial depth and nutrient levels (*P* < 0.05; **Table 1**). When growing in low-nutrient sediment, the aboveground biomass of *C. thunbergii* was higher in the 3 cm sediment layer than in the 6 cm sediment layer (**Figure 3E**). Nutrient addition increased the aboveground biomass of *C. thunbergii* growing in the 6 cm sediment burial treatment (**Figure 3E**).

**Figure 4:**
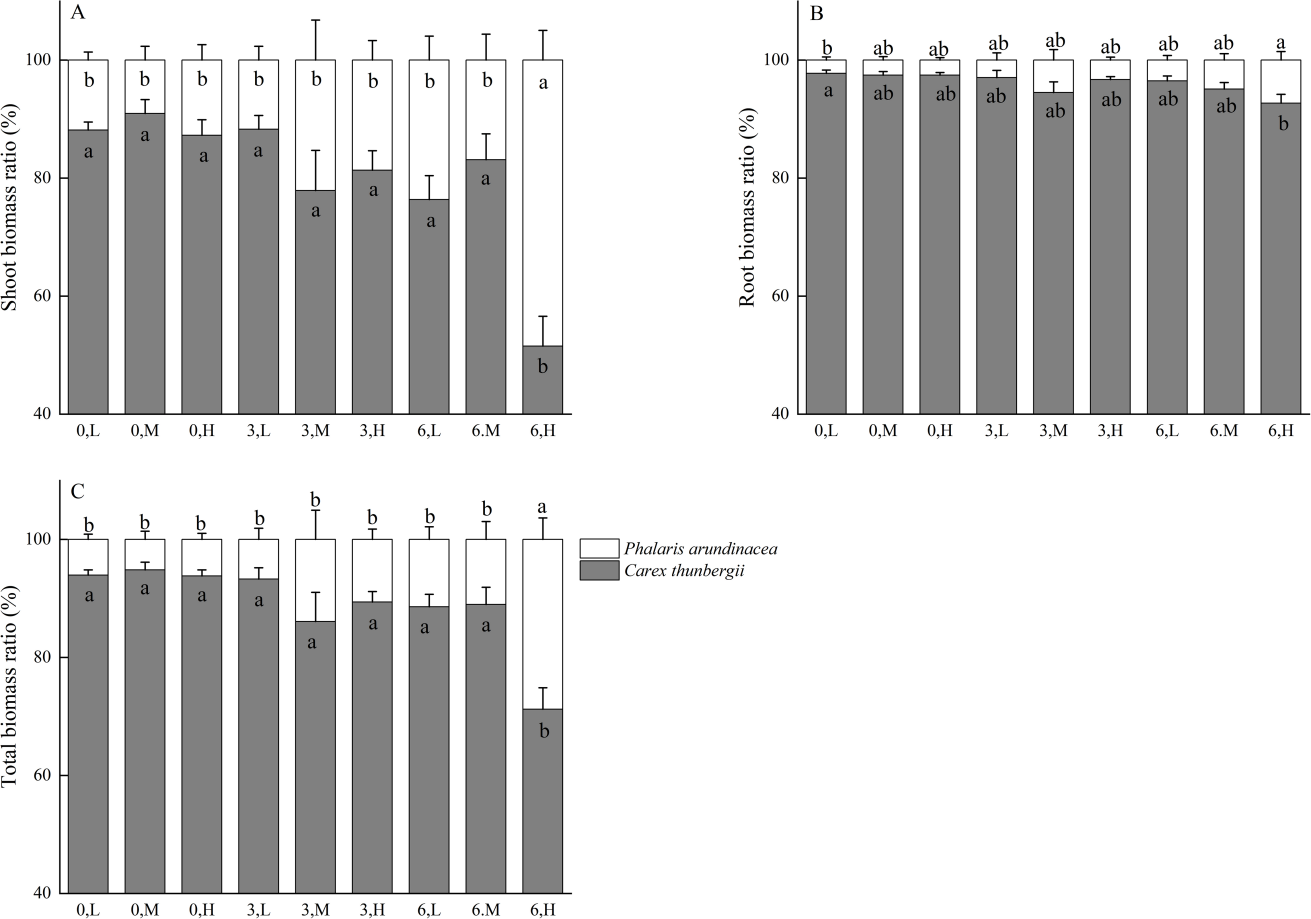
Shoot, root, and total mass ratio of *Phalaris/Carex* at three burial depths and three nutrient levels **(A–C)**. Different lowercase letters indicate significant differences among the treatments (P < 0.05). Treatments: 0 cm burial depth with low nutrients (0, L); 0 cm burial depth with medium nutrients (0, M); 0 cm burial depth with high nutrients (0, H); 3 cm burial depth with low nutrients (3, L); 3 cm burial depth with medium nutrients (3, M); 3 cm burial depth with high nutrients (3, H); 6 cm burial depth with low nutrients (6, L); 6 cm burial depth with medium nutrients (6, M); and 6 cm burial depth with high nutrient levels (6, H).

The ratio of shoot biomass of *P. arundinacea* to the shoot biomass of *C. thunbergii* ranges from 9.04% to 48.47% across treatments (**Figure 4A**). The ratio of root biomass of *P. arundinacea* to the root biomass of *C. thunbergii* ranges from 2.23% to 7.28% across treatments (**Figure 4B**). The ratio of total biomass of *P. arundinacea* to the total biomass of *C. thunbergii* ranges from 5.17% to 28.78% across treatments (**Figure 4C**). The ratios of the shoot, root, and total biomass of *P. arundinacea* to the shoot, root, and total biomass of *C. thunbergii* were higher in the 6 cm sediment burial treatment with high nutrient addition than that in the 0 cm burial depth treatment without nutrient addition (**Figures 4A–C**).

### 3.2 Ramet height and root length

**Figure 5:**
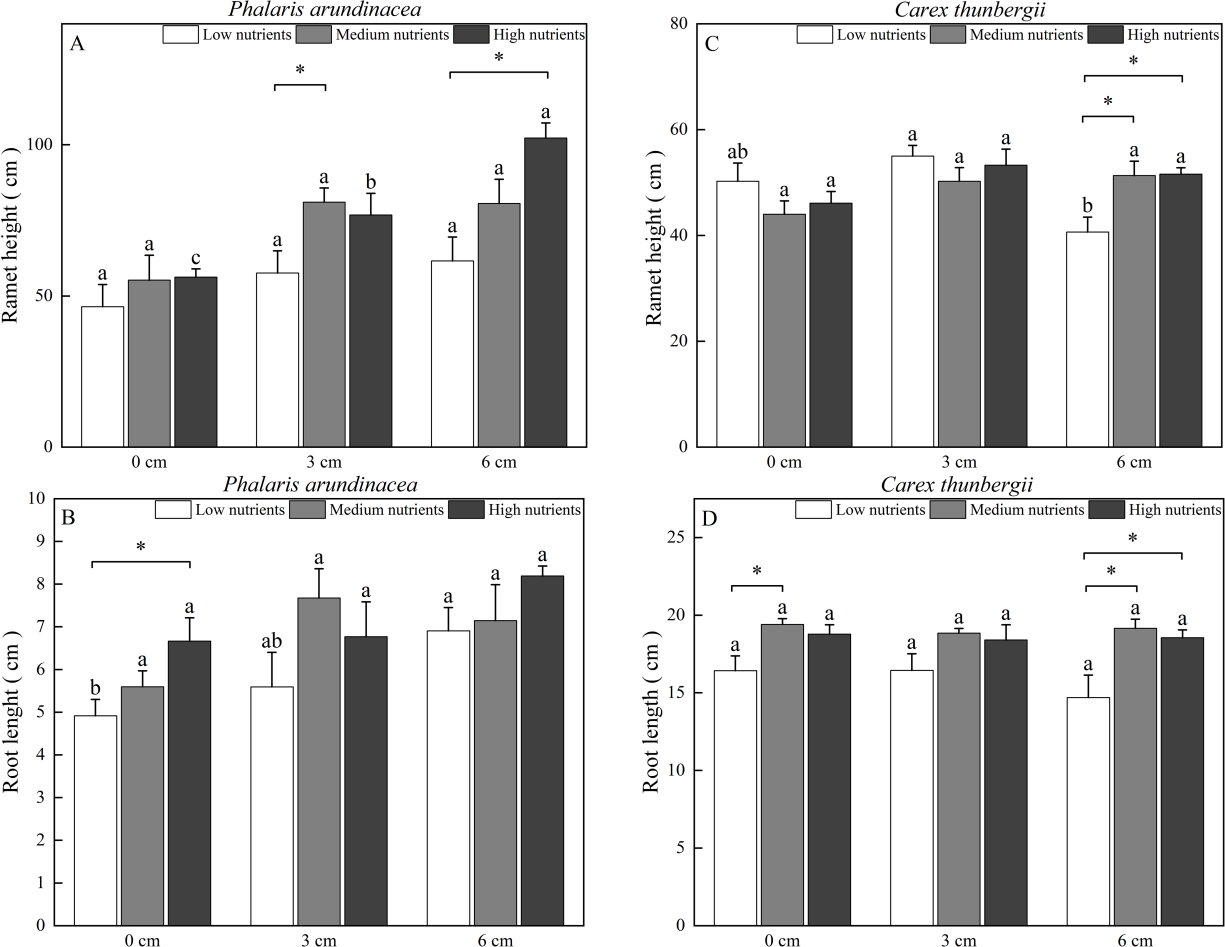
Ramet height and root length of Phalaris arundinacea **(A, B)** and *Carex thunbergii* **(C, D)** growing at three sediment burial depths with three nutrient levels. Different lowercase letters indicate significant differences among burial depth treatments at the same nutrient level (P < 0.05). * indicates significant differences among different nutrient level treatments at the same sediment burial depth (P < 0.05).

The ramet height of *P. arundinacea* was affected by burial depth and nutrient levels (*P* < 0.05; **Table 1**). When growing in high-nutrient sediments, the ramet height of *P. arundinacea* increased with sediment depth. Nutrient addition increased the ramet height of *P. arundinacea* in both the 3 and 6 cm burial depth treatments (**Figure 5A**). The ramet height of *C. thunbergii* was affected by burial depth and had a significant interaction with nutrient levels (**Table 1**). Without nutrient addition, the ramet height of *C. thunbergii* was shorter in the 6 cm burial depth treatment than that in the 3 cm burial depth treatment (**Figure 5C**). At a sediment burial depth of 6 cm, addition of nutrients increased the ramet height of *C. thunbergii* (**Figure 5C**).

The root length of *P. arundinacea* was affected by burial depth and nutrient levels (*P* < 0.05; **Table 1**). Without nutrient addition, the root length of *P. arundinacea* was greater in the 6 cm sediment burial treatment than that in 0 cm burial depth treatment (**Figure 5B**). Without sediment burial, nutrient addition increased the root length of *P. arundinacea* (**Figure 5B**). The root length of *C. thunbergii* was only affected by nutrient levels (**Table 1**). At a sediment burial depth of 6 cm, nutrient addition increased the root length of *C. thunbergii* (**Figure 5D**).

### 3.3 Number of rhizomes and ramets

**Figure 6:**
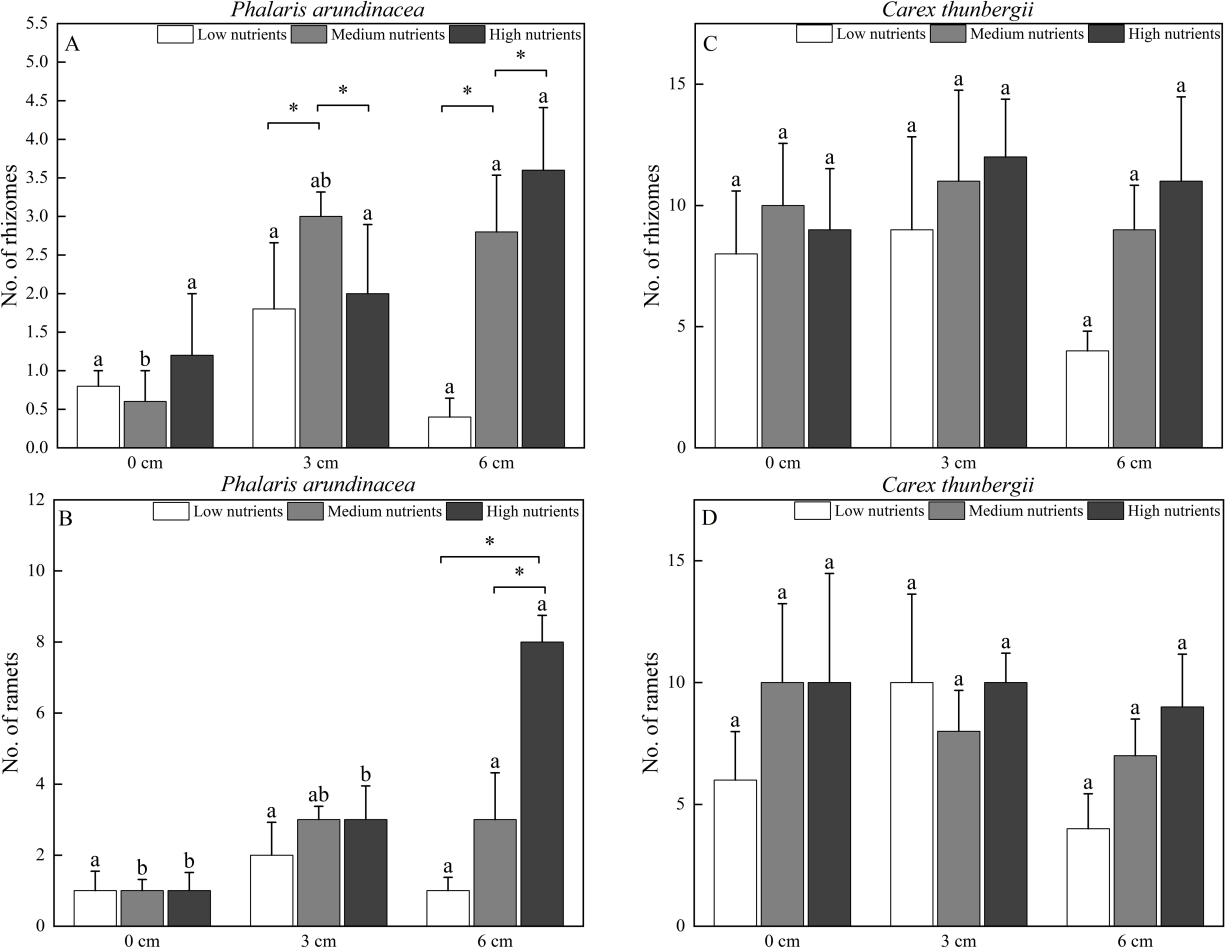
The number of rhizomes and ramets of Phalaris arundinacea **(A, B)** and *Carex thunbergii* **(C, D)** growing at three sediment burial depths with three nutrient levels. Different lowercase letters indicate significant differences among burial depth treatments at the same nutrient level (P < 0.05). * indicates significant differences among different nutrient level treatments at the same sediment burial depth (P < 0.05).

The number of rhizomes produced by *P. arundinacea* was significantly affected by the sediment burial depth, with significant interactions with nutrient levels (*P* < 0.05; **Table 1**). When grown under medium nutrient conditions, *P. arundinacea* produced more rhizomes in the 6 cm burial depth treatment than in the 0 cm burial depth treatment (**Figure 6A**). Nutrient addition increased the number of rhizomes produced by *P. arundinacea* when subjected to 3 and 6 cm sediment burial (**Figure 6A**). The number of rhizomes and ramets produced by C. thunbergii was neither affected by burial depth or nutrient levels (**Figures 6C, D**).

The number of ramets produced by *P. arundinacea* was significantly affected by burial depth and nutrient levels (*P* < 0.05; **Table 1**). With the addition of nutrients, the number of ramets produced by *P. arundinacea* was higher in the 6 cm burial depth treatment than in the 0 cm burial depth treatment (**Figure 6B**). At a burial depth of 6 cm, addition of nutrients increased the number of ramets produced by *P. arundinacea* (**Figure 6B**). The number of ramets produced by *C. thunbergii* was neither affected by burial depth or nutrient levels (**Table 1**; **Figure 6D**).

## 4 Discussion

### 4.1 High sediment accretion inhibited *C. thunbergii* growth but did not affect *P. arundinacea*

In our study, 6 cm sediment burial without nutrient addition decreased the aboveground and total biomass of *C. thunbergii* but had no effect on *P. arundinacea* (**Figures 3A–D**). As a non-stem sedge, *C. thunbergii* can escape sediment burial by producing spreading ramets via rhizome elongation (Li et al., 2015). However, projecting new ramets onto a sediment surface consumes large quantities of carbohydrate reserves (Pan et al., 2012; Chen et al., 2017b). Consequently, the biomass of *C. thunbergii* decreases with heavy sediment burial. In contrast, *P. arundinacea* can escape sediment burial through rapid stem growth and internode elongation (Chen et al., 2014, 2017a). Therefore, *P. arundinacea* can tolerate higher sediment accretion than *C. thunbergii*, which is consistent with hypothesis 1. Hypothesis 1 was also supported by the higher proportional biomass of *P. arundinacea* to *C. thunbergii* in the heavy sedimentation treatment (**Figure 4C**).

### 4.2 Moderate sedimentation enhanced *P. arundinacea* growth but did not affect *C. thunbergii*

Our results showed that moderate sediment burial with nutrients enhanced the aboveground and total biomass of *P. arundinacea* but had no effect on *C. thunbergii*. Studies have indicated that moderate sedimentation stimulates macrophyte growth (Maun, 1998; Gilbert and Ripley, 2010; Chen et al., 2017a; Fan et al., 2018). For example, moderate sedimentation increases the growth of *Spartina patens*, *Scirpus mariqueter*, and *S. alterniflora* (Halun et al., 2002; Matzke and Elsey-Quirk, 2018; Xiao et al., 2023). The stimulation of macrophyte growth upon sedimentation may be attributed to increased soil volume, nutrients, moisture, and microbial activity (Maun, 1998). For *P. arundinacea*, increased nutrient availability may have been the primary contributor stimulating growth during sediment burial (Katagiri et al., 2011; Chen et al., 2017a).

Nevertheless, we did not observe a stimulatory effect of moderate sedimentation on *C. thunbergii* growth. Non-caulescent *Carex* species adapt to sedimentation by changing their clonal growth from economically clumped ramets to costively spreading ramets (Bernard, 1990; Chen et al., 2010; Li et al., 2015). Even with moderate sediment burial (3 cm), the proportion of spreading ramets produced by *C. brevicuspis* increased significantly, indicating that *Carex* species were sensitive to sediment burial. Furthermore, the increased growth of *P. arundinacea* after sediment deposition may have suppressed the growth of native species through light interception (Kercher and Zedler, 2004). *Phalaris arundinacea* can effectively utilize resources such as light and sediment nutrients to produce additional ramets, enhancing its competitive capacity (Martina and von Ende, 2012; Chen et al., 2017a; Winikoff et al., 2020). Therefore, *P. arundinacea* may have a growth advantage over *C. thunbergii* under moderate sedimentation conditions.

### 4.3 Nutrient enrichment increased the vegetative propagation of *P. arundinacea* but not *C. thunbergii*

Our results indicated that high sediment accretion with nutrients increased the number of rhizomes and ramets produced by *P. arundinacea* but did not affect *C. thunbergii*. This result is consistent with hypothesis 2: *P. arundinacea* benefits more from nutrient enrichment than *C. thunbergii* does. Other studies also found that nutrient addition increased the abundance and production of *P. arundinacea* in wet prairie assemblages during *P. arundinacea* invasion (Kercher and Zedler, 2004; Weston et al., 2021). As an opportunistic invader, *P. arundinacea* may efficiently utilize fluctuating resources and produce additional ramets to occupy its habitat (Davis et al., 2000; Kettenring et al., 2019). In contrast, *Carex* species may adopt a conservative reproductive strategy to manage resource availability, that is, produce a relatively constant number of rhizomes and ramets but change individual size (Chen et al., 2016). Therefore, with increasing nutrient input in floodplain wetlands, the abundance of *P. arundinacea* may increase in *Carex* grasslands.

## 5 Conclusion

Our study indicated that high sediment accretion inhibited the growth of *C. thunbergii* but had no effect on *P. arundinacea*. Moderate sedimentation enhanced the growth of *P. arundinacea* but did not affect *C. thunbergii*. Nutrient enrichment of sediments increased the vegetative propagation of *P. arundinacea* but did not affect *C. thunbergii*. From these results, we inferred that in floodplain wetlands, *P. arundinacea* may increase in abundance and coverage when growing in *Carex* grasslands subjected to sediment accretion and nutrient enrichment. Therefore, to maintain the ecological function of floodplain wetlands, management measures should be implemented to reduce sediment load and nutrient input to protect *Carex* grasslands from *P. arundinacea* invasion.

## Data Availability

The raw data supporting the conclusions of this article will be made available by the authors, without undue reservation.
